# New Insights into the Immunobiology of Mononuclear Phagocytic Cells and Their Relevance to the Pathogenesis of Cardiovascular Diseases

**DOI:** 10.3389/fimmu.2017.01921

**Published:** 2018-01-09

**Authors:** Liliana Maria Sanmarco, Natalia Eberhardt, Nicolás Eric Ponce, Roxana Carolina Cano, Gustavo Bonacci, Maria Pilar Aoki

**Affiliations:** ^1^Departamento de Bioquímica Clínica, Facultad de Ciencias Químicas, Universidad Nacional de Córdoba, Córdoba, Argentina; ^2^Centro de Investigaciones en Bioquímica Clínica e Inmunología (CIBICI), Consejo Nacional de Investigaciones Científicas y Tecnológicas (CONICET), Córdoba, Argentina; ^3^Laboratorio de Neuropatología Experimental, Instituto de Investigación Médica Mercedes y Martín Ferreyra (INIMEC), Consejo Nacional de Investigaciones Científicas y Tecnológicas (CONICET), Universidad Nacional de Córdoba, Córdoba, Argentina; ^4^Consejo Nacional de Investigaciones Científicas y Tecnológicas (CONICET), Universidad Católica de Córdoba, Unidad Asociada Área Ciencias Agrarias, Ingeniería, Ciencias Biológicas y de la Salud, Facultad de Ciencias Químicas, Córdoba, Argentina

**Keywords:** macrophages, monocytes, Chagas disease, atherosclerosis, oxidative stress, interleukin-6, oxidized phospholipids, purinergic signaling

## Abstract

Macrophages are the primary immune cells that reside within the myocardium, suggesting that these mononuclear phagocytes are essential in the orchestration of cardiac immunity and homeostasis. Independent of the nature of the injury, the heart triggers leukocyte activation and recruitment. However, inflammation is harmful to this vital terminally differentiated organ with extremely poor regenerative capacity. As such, cardiac tissue has evolved particular strategies to increase the stress tolerance and minimize the impact of inflammation. In this sense, growing evidences show that mononuclear phagocytic cells are particularly dynamic during cardiac inflammation or infection and would actively participate in tissue repair and functional recovery. They respond to soluble mediators such as metabolites or cytokines, which play central roles in the timing of the intrinsic cardiac stress response. During myocardial infarction two distinct phases of monocyte influx have been identified. Upon infarction, the heart modulates its chemokine expression profile that sequentially and actively recruits inflammatory monocytes, first, and healing monocytes, later. In the same way, a sudden switch from inflammatory macrophages (with microbicidal effectors) toward anti-inflammatory macrophages occurs within the myocardium very shortly after infection with *Trypanosoma cruzi*, the causal agent of Chagas cardiomyopathy. While in sterile injury, healing response is necessary to stop tissue damage; during an intracellular infection, the anti-inflammatory milieu in infected hearts would promote microbial persistence. The balance of mononuclear phagocytic cells seems to be also dynamic in atherosclerosis influencing plaque initiation and fate. This review summarizes the participation of mononuclear phagocyte system in cardiovascular diseases, keeping in mind that the immune system evolved to promote the reestablishment of tissue homeostasis following infection/injury, and that the effects of different mediators could modulate the magnitude and quality of the immune response. The knowledge of the effects triggered by diverse mediators would serve to identify new therapeutic targets in different cardiovascular pathologies.

## Introduction

Cardiovascular diseases are the leading causes of worldwide morbidity and mortality. In consequence, understanding the precise contribution of the mechanisms involved in cardiovascular tissue injury and repair is of prominent importance. In this sense, increasing evidences reveal that innate immune response plays a critical and complex role throughout the acute inflammation and regenerative process triggered after cardiac or vascular injury. As such, leukocytosis and monocytosis have been associated with cardiovascular diseases in numerous epidemiological studies, prompting speculation on the functional importance of these cells (1). The goal of this review is to summarize the complex immunobiology of mononuclear phagocytic cells and their relevance to the pathogenesis of cardiovascular diseases, highlighting the effect of major immune modulators.

### Origin

In the last decade, important advances in the knowledge of macrophage origin have triggered an essential conceptual progress in the mononuclear phagocyte system field. Parabiosed mice and genetic fate-mapping experiments have revealed that the majority of resident macrophages in healthy tissues are established from the yolk sac and fetal liver before birth (2–4). This cellular compartment locally self-maintains throughout life within the tissue and is independent from the hematopoietic input. On the other hand, during adulthood tissue-infiltrating macrophages can develop from circulating monocytes. The recruitment of monocytes is associated with pathological, but also with homeostatic response. Macrophages derived from monocytes display a short lifespan, although exceptions have also been reported. Embryonic- and adult-derived macrophages generally coexist in a given tissue, and their respective number correlates with the origin and records of their tissue of residence. Seminal experiments have demonstrated that embryonic- and monocyte-derived macrophages make different functional contributions in homeostatic conditions or following challenge. In this sense, Levine and coworkers have revealed that embryonic-derived macrophages are clue for cardiac recovery after injury (5). Their results suggest that targeting specific macrophage lineages could have important therapeutic implications in order to improve treatments for heart diseases.

Adult resident macrophage compartments seem to be independent from monocyte recruitment in a given tissue, as was demonstrated by parabiotic experiments. Monocyte compartments of the parabionts reach, with time, considerable chimerism in the joined circulation. However, tissue macrophages failed to equilibrate even after several months of parabiosis, suggesting the absence of an ongoing steady-state contribution of bone marrow-derived cells to adult tissue macrophage compartments (3). Furthermore, recruited monocytes are short-lived effector cells in tissues and assumed different roles that have yet to be better defined; emerging reports suggest, for example, that monocytes can promote angiogenesis and arteriogenesis (6). In addition, it was recently reported that inflammatory Ly6C^high^ monocytes persist during the steady state without commitment toward macrophage or dendritic cell (DC) fates and might contribute to antigen transport toward lymph nodes (7). Remarkably, macrophage effector function also needs to be tailored to its tissue of residence, an adaptation that is driven by the local microenvironment and by the inflammatory history of a given tissue.

### Phenotypes and Functions of Monocytes

Monocytes/macrophages are very plastic cells and can acquire distinct phenotypes and activation states under the influence of different microenvironments. Although several authors have shown that macrophages treated with different stimuli display altered phenotypes or functional capacities, many of these studies are limited considering that they compare only one particular activation state with non-polarized macrophages. Although macrophage activation was initially seen as a dichotomy between classically and alternatively activated states, it is now clear that the spectrum of macrophage activation states is much more diverse.

**Figure 1:**
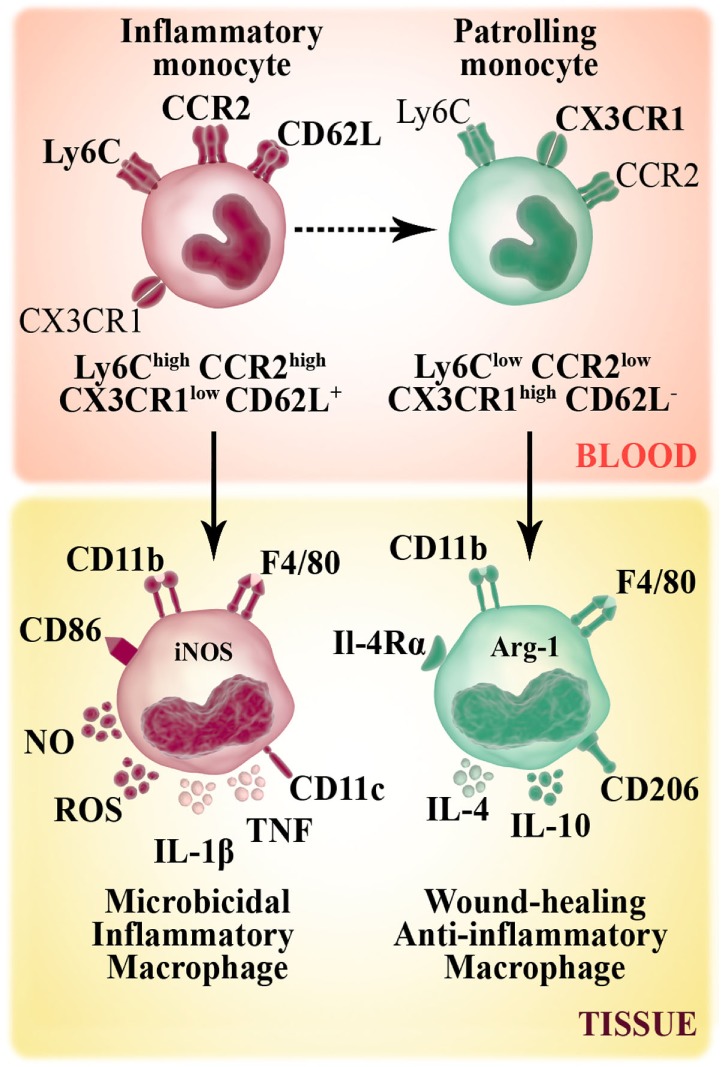
Murine monocyte and macrophage subsets. Murine monocytes develop from a common myeloid progenitor and leave the bone marrow through the chemokine receptor CCR2. In bloodstream, circulating monocytes are phenotypically and functionally heterogeneous. Non-classical monocytes (Ly6C^low^ CCR2^low^ CX3CR1^high^ CD62L^−^) patrol the vasculature and accumulate at low numbers in the steady state. Inflammatory or classical (Ly6C^high^ CCR2^high^ CX3CR1^low^ CD62L^+^) monocytes have a relatively short-circulating lifespan and preferentially accumulate in inflammatory sites where they give rise to inflammatory M1 macrophages (F4/80^+^ CD11b^+^ CD86^+^ CD206^−^). M1 macrophage subset has high microbicidal capacity due to their ability to produce inflammatory cytokines [TNF, IL-1β], reactive oxygen species (ROS) secretion and the expression of iNOS enzyme that metabolizes arginine to arginine-derived killer molecule NO. Non-classical monocytes can be recruited to tissue and differentiate to M2 macrophages (F4/80^+^ CD11b^+^ CD86^−^ CD206^+^), which secrete anti-inflammatory cytokines (IL-10, IL-4) and contribute to tissue repair mechanisms.

Monocytes develop in steady state in the bone marrow from hematopoietic precursors, and they enter the circulation *via* CCR2 receptor. In mice, circulating monocytes are phenotypically and functionally heterogeneous and can be defined according to Ly6C expression marker (Figure 1). In mice, during steady-state conditions, about 50–60% of circulating monocytes belongs to the Ly6C^high^ CCR2^high^ CX3CR1^low^ CD62L^+^ subset. These inflammatory or classical monocytes have a relatively short-circulating lifespan and are preferentially recruited to injured/inflamed tissues where they maturate to macrophages. The remaining non classical (Ly6C^low^ CCR2^low^ CX3CR1^high^ CD62L^−^) subset patrols blood vessels and accumulates at low numbers in the steady state (8). The number of Ly6C^high^ monocytes rises during inflammation, at expenses of enhanced monocytopoiesis in the bone marrow and spleen (1, 9–11). Regarding the origin, evidence shows that monocyte subpopulations do not arise from separate progenitors, but rather convert from the Ly6C^high^ to Ly6C^low^ subset (12) (Figure 1).

**Figure 2:**
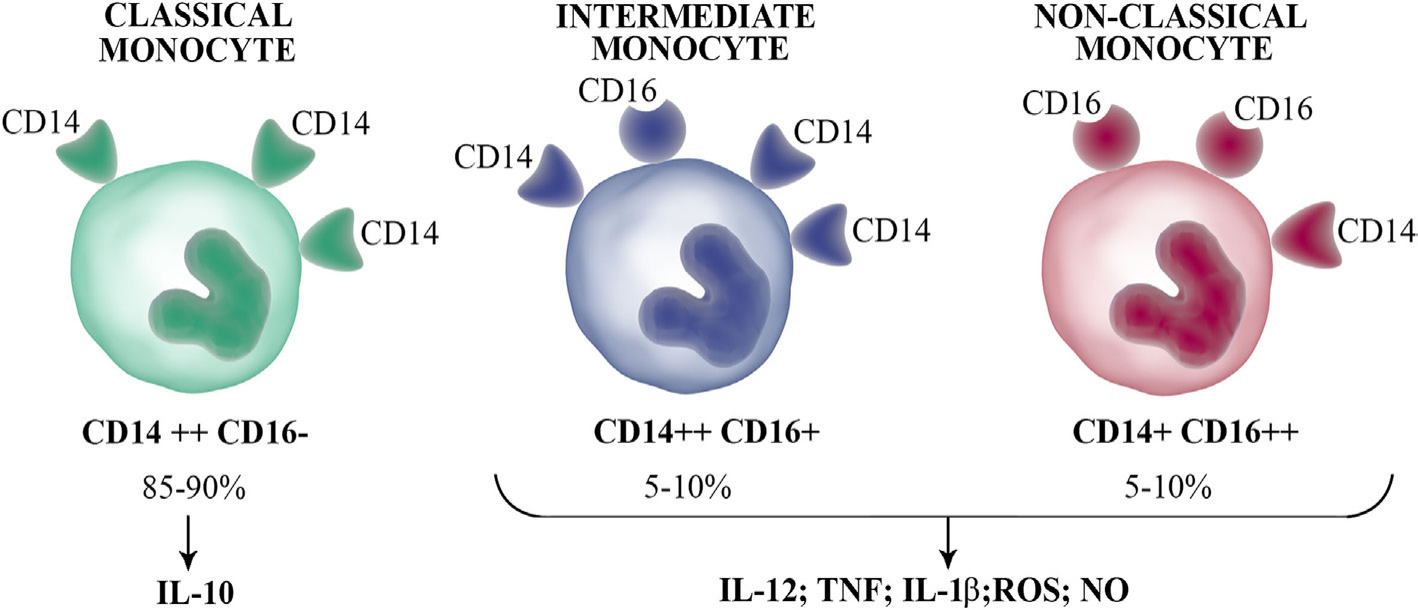
Human circulating monocyte subsets. Human blood monocytes can be separated into three subsets according to the CD16 and CD14 expression: classical monocytes (CD14^++^ CD16^−^), which represent the majority of circulating monocytes produce the anti-inflammatory IL-10 upon stimulation; intermediate monocytes (CD14^++^ CD16^+^); and non-classical monocytes (CD14^+^ CD16^++^), which secrete inflammatory cytokines such as IL-1β, IL-12, TNF, and antimicrobial molecules [nitric oxide (NO) and reactive oxygen species (ROS)].

In humans, circulating monocytes can be segregated into three major subsets based on the expression of CD14 and CD16 (13). Over the past decades, human circulating monocytes had been separated into two subpopulations based on CD16 expression, the CD14^+^ CD16^−^ and CD14^+^ CD16^+^ monocyte subsets (hereby designated as CD16^−^ and CD16^+^, respectively). A new nomenclature defines three monocyte populations, where the minor CD16^+^ subset is further separated into two subpopulations (Figure 2). The intermediate subset expresses relatively high levels of CD14 coupled with low CD16 expression (CD14^++^ CD16^+^), while the non-classical subset expresses low levels of CD14 with high expression of CD16 (CD14^+^ CD16^++^). The subset with high CD14 and no CD16 expression, termed classical subset (CD14^++^ CD16^−^), constitute approximately 85–90% of human monocytes in the peripheral blood. Several studies have demonstrated that circulating CD16^+^ monocytes are found in large numbers in patients with inflammation processes (14) and infectious diseases (15, 16).

Analyses based on hierarchical clustering of gene expression profiles of human monocyte subsets have been a matter of debate. Cros et al. reported that the classical and intermediate subpopulations were most closely related among the subsets, while the most distant was the non-classical subset (17). However, other independent groups described that both CD16^+^ subpopulations are more closely related (18, 19). Indeed, the number of genes that were significantly different between both CD16^+^ subsets was the lowest among the three subpopulations. Until now, there is poor agreement on the effector functions of the three monocyte subsets. In this sense, it was reported that TNF is produced mainly by CD16^+^ monocytes (intermediate and non-classical subsets) (20, 21). However, recent results have evidenced that TNF is produced by the three subsets. While Cros et al. (17) described that non-classical monocytes were poor producers of several cytokines [TNF, interleukin (IL)-1β, CCL2, IL-10, IL-8, IL-6, and CCL3] in response to LPS, other reports showed that the stimulation of intermediate subset with LPS produced the most TNF, IL-1β, and IL-6. In agreement, others also showed that intermediate monocytes produced the most TNF and IL-1β upon treatment with LPS and that this subset is the highest producer of TNF when cocultured with pre-activated T cells (22). In addition, Wong et al. (18) showed that non-classical monocytes upon LPS stimulation produced the highest levels of TNF and IL-1β, while equivalent amounts of IL-6 and IL-8 were produced by all three subsets. Other reports also recognized non-classical monocyte population as the greatest producer of TNF (21). Hence, it seems that non-classical monocytes constitute the subset capable of producing inflammatory cytokines in response to TLR ligands. There are also controversies on which monocyte subset is the major producer of the anti-inflammatory cytokine IL-10. In this sense, although it was demonstrated that intermediate monocytes produced the most IL-10 in response to LPS and zymosan (23), some recent reports showed that classical monocytes produced the most IL-10 (17, 18, 24). Further studies are necessary to clarify this relevant question.

### Phenotypes and Functions of Macrophages

Several studies on macrophage biology have focused on the particular functions that these cells acquire after tissue accumulation. Macrophages are highly plastic and can adapt to environmental stimuli, displaying either a classically activated (M1) or alternatively activated (M2) profile, which represent extremes of a spectrum of functional phenotypes (25, 26). M1 macrophages are activated by pro-inflammatory Th1 cytokines and LPS. Besides being antigen-presenting cells, the M1 subset shows an enhanced microbicidal capacity attributable to the production of reactive oxygen species (ROS) (such as hydrogen peroxide, superoxide, nitric oxide (NO), and peroxynitrite) and inflammatory cytokines (TNF, IL-1β, IL-12, and IL-23). On the other extreme, M2 macrophages are activated by Th2 cytokines (IL-4 and IL-13) or by anti-inflammatory mediators (IL-10), enhancing the arginase activity and mannose receptor (CD206) expression, to promote wound healing and reduce Th1 response. However, the M2 subset development can also be detrimental to host tissue, leading to fibrosis when their matrix-enhancing activity is not regulated (27). Although embryonic and monocyte-derived macrophages likely are on a continuum spectrum that lies between (and outside of) the M1 and M2 classifications, the terminology nevertheless has been helpful in order to elucidate macrophage heterogeneity. When Ly6C^high^ monocytes are recruited to atherosclerotic lesions, they mature to F4/80^+^ macrophages. In a persistent inflammatory environment, these Ly6C^high^ monocyte-derived macrophages contribute to oxidative stress and are inflammatory by producing IL-1β and TNF (9). In this respect, Ly6C^high^ monocyte-derived cells are M1 macrophages. In the setting of inflammation resolution, M1 macrophages are replaced by M2 repairing macrophages. Although it has been proposed that M1 to M2 conversion occurs locally (28–30), M2 macrophages also could derive from non-classical and less inflammatory Ly6C^low^ monocytes (12). Otherwise, M2 macrophages may arise through direct differentiation of Ly6C^high^ monocytes in a microenvironment that favors wound healing. This option should be further explored because it is enticing for potential therapeutic reasons.

Cardiac resident macrophages coexpress M1 and M2 markers suggesting no specific polarization (31). However, within myocardium macrophages respond to systemic Th2 environment induced by helminth parasites infection, adopting an M2 phenotype associated with enhanced fibrosis. In this model, the increased amount of cardiac macrophages relies on recruitment of Ly6C^high^CCR2^+^ monocytes instead of IL-4-induced expansion. In this sense, Jenkins et al. have shown that IL-4 and IL-13 through IL-4Rα not only activate macrophages but also cause proliferative expansion of resident macrophages (32, 33). Moreover, IL-33 also induces macrophages proliferation, but in an IL-4Rα-signaling-independent manner (34), suggesting that the number and activation state of cardiac macrophages largely depend on mediators locally produced.

## Relevance to Cardiovascular Pathologies

Mononuclear phagocytes are becoming increasingly recognized as key cells in the initiation and propagation of cardiac injury and remodeling. This growing body of evidence should prompt cardiovascular researchers to explore new therapeutic targets. Because of their functional and phenotypic versatility, manipulating specific macrophage subsets may spare key and vital cardiovascular functions, such as tissue repair and defense against pathogens, while preventing specific deleterious effects that contribute to adverse cardiac remodeling or atherosclerotic plaque formation.

### Infections

#### Chagas Cardiomyopathy

Chagas disease is caused by *Trypanosoma cruzi* infection and constitutes a major public health problem in Latin America due to its prevalence, morbidity, and mortality. The World Health Organization (WHO) classifies it as a neglected tropical disease, and has recently estimated that 6–7 million people worldwide are infected, and about 7,000 people annually die (35). The cardiomyopathy represents the most frequent and serious complication of Chagas disease, affecting about 30–40% of infected individuals. In consequence, Chagas myocarditis is the most common form of infectious cardiomyopathy worldwide.

The clinical course of the disease is divided into acute and chronic phases. During the acute phase, which lasts 2–4 months, the infective trypomastigote form is in the bloodstream and invades target cells, where it replicates as amastigote form. This stage is followed by a life-long chronic phase, in which parasites are cleared from the circulation but persist for years within the myocardium among other target tissues. Nowadays, it is widely accepted that the persistence of parasites in target tissues coupled with an unbalanced immune response is a necessary and sufficient condition for the development of the cardiomyopathy (36–39).

In response to the infection, macrophages are one of the main infiltrating leukocytes arriving early to the myocardium (40) and they remain as an important immune cell population in heart explants from patients with severe advanced chronic Chagas disease (41). Interestingly, selective depletion of macrophages causes a significant increment in the number of amastigote nests within cardiomyocytes, suggesting a crucial role for macrophages in the resistance to this infection (42). Even though several anti-parasitic roles of these cells in Chagas disease has been further documented, the functional characterization of resident and infiltrating macrophages in infected myocardium has not been widely studied until very recently. In this sense, we have described the kinetics of macrophage populations during the acute and chronic phase of *T. cruzi* infection in BALB/c and C57BL/6 cardiac tissue (43, 44). In both mouse strains, macrophages with M1 phenotype (CD45^+^ F4/80^+^ CD11b^+^ CD86^+^ CD206^−^) predominate only at short times postinfection [4 days postinfection (dpi)], but then macrophages are rapidly polarized toward M2 phenotype (CD45^+^ F4/80^+^ CD11b^+^ CD86^−^ CD206^+^), which remains sustained during the acute and chronic phase (90 dpi). The sudden shift in cardiac M1/M2 ratio correlates with the changes in local cytokine milieu. These works evidence that alternatively activated macrophages are the main cardiac cell subset throughout the infection. Alternative macrophages provide protection against overwhelming cardiac inflammation, but they also interfere with the activation of M1 macrophages and its microbicidal function, promoting parasite persistence. The sustained M2 profile is promoted, at least in part, by the action of IL-6 (44).

**Figure 3:**
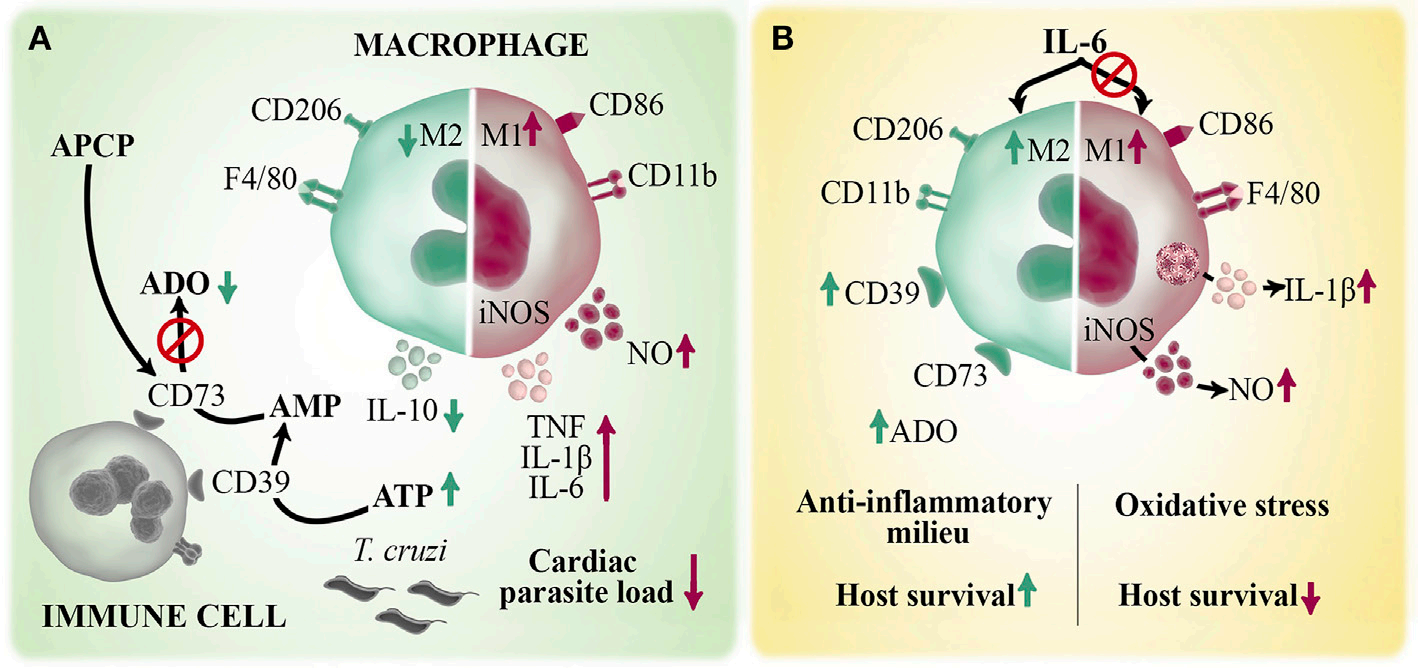
Role of macrophages in the immune response against *Trypanosoma cruzi* infection. Macrophages are the main infiltrating cells arriving to the myocardium early after *T. cruzi* infection. Different mediators can regulate the magnitude and quality of the cardiac immune response by modulating macrophages activation. **(A)** ATP is released by infected/injured cells and hydrolyzed by two ectoenzymes, CD39 and CD73, to the immunoregulatory metabolite ADO. Pharmacological inhibition of CD73 activity with APCP enhances M1 over M2 macrophage phenotype and increases the local production of TNF, IL-1β, IL-6, and NO and diminished IL-10 levels. **(B)** IL-6 is a pleiotropic cytokine that contributes to the establishment of cardiac M2 macrophage profile during *T. cruzi* infection by inducing an anti-inflammatory microenvironment and augmented CD39 expression. Deficiency of IL-6 (IL6KO mice) dysregulates inflammasome activation with a consequent increases in IL1-β-induced NO production. The excessive oxidative stress and exacerbated pro-inflammatory immune response cause the lethal effect observed in infected IL6KO mice.

In order to stablish the possible mechanisms by which cardiac M1 macrophages shift toward M2 profile quickly after infection, we focused on purinergic signaling. The ecto-5′-nucleotidase (CD73) enzyme is part of the purinergic system and together with ectonucleoside triphosphate diphosphohydrolase-1 (CD39) metabolize, in a step-wise manner, the extracellular ATP into adenosine (ADO). ADO has a regulating role on macrophage functions associated with M1 profile, such as suppression of chemokines and pro-inflammatory cytokines production, impairment of inducible nitric oxide synthase (iNOS) activity and induction of anti-inflammatory IL-10 production (45). CD73 is the rate-limiting enzyme in the ADO generation and it is expressed by immune and parenchymal cells (46). We showed that pharmacological inhibition of CD73 enzymatic activity with ADO 5′α,β-methylene-diphosphate (APCP) during the early acute phase enhances the number of M1 cardiac macrophages over the M2 subset and increases cardiac levels of NO, IL-6, TNF, and IL-1β in infected BALB/c mice (Figure 3A). These APCP-induced changes result in a concomitant reduction of cardiac parasite load during the acute phase and, as a direct consequence, improve the outcome of chronic cardiomyopathy (43). The study demonstrates that the polarization of cardiac macrophages is strongly influenced by the products of extracellular ATP metabolism and that the manipulation of this pathway could provide new approaches to reduce Chagas heart pathology.

In response to different cardiac injuries, soluble mediators are key players since they drive the immune response and the reestablishment of homeostasis. In this sense, IL-6 cytokine is suddenly and continuously produced by cardiomyocytes and neighboring cells after different pathological stimuli, suggesting that this cytokine is clue for the heart’s intrinsic stress response system. We and others have reported that IL-6-deficient (IL6KO) mice succumb to *T. cruzi* infection very early during the acute phase (44, 47, 48). By analyzing immune mechanism that could explain the lethal effect observed in the absence of this cytokine, we discovered that IL-6 negatively regulates inflammasome activation and, consequently, IL-1β-induced NO production, and that excessive oxidative stress accounts for the increased mortality of infected IL6KO mice (44). We also found that IL-6 promotes the establishment of M2 cardiac macrophage profile during infection and induces *in vivo* and *in vitro* expression of the enzyme CD39 on macrophages, suggesting that IL-6 could promote a shift from an ATP driven pro-inflammatory environment to an anti-inflammatory milieu induced by ADO (44) (Figure 3B). Considering that IL-6 is a therapeutic target in individuals with different inflammatory diseases (49), our results provide the basis for understanding why blocking IL-6 in certain clinical situations does not represent an effective treatment; instead triggering pro-inflammatory adverse events.

In agreement with the results obtained with the mouse experimental model, the culture of *in vitro*-infected peripheral blood mononuclear cells obtained from control human donors with IL-6 blunted *T. cruzi*-induced nitration of cytotoxic T-cell subpopulation and increased their survival. The nitration of surface proteins on T cells is a consequence of NO and superoxide anion reaction which generates peroxynitrite. Furthermore, the blockade of IL-6 in these cultures with an IL-6 neutralizing antibody increased the frequency of *T. cruzi*-induced nitration of CD8^+^ T cells as a consequence of elevated NO production. In line with these results, leukocytes from chronic Chagas patients show increased NO production concomitant with significant tyrosine nitration mainly in CD8^+^ T cells compared with seronegative patients. Superficial tyrosine nitration on cytotoxic cells is concomitant with impaired effector functions and lower capacity for activation but also it is associated with a significant fall in the number of circulating CD8^+^ T cells (50). Altogether, the results suggest that CD8^+^ T cell functionality decreases in the setting of human Chagas disease, but this under-responsiveness could be reverted by the pleiotropic actions of IL-6, which functions as a survival factor for this population and improves its effector functions. Strikingly, the parasite has evolved strategies to counteract IL-6-dependent signaling through the specific cleavage of its receptor, the gp130 chain (51).

In contrast to the antioxidant properties of IL-6, IFN-γ enhances iNOS expression in infected macrophages resulting in the killing of intracellular parasites. Strikingly, IL-17A-stimulated macrophages control replication in a comparable level to IFN-γ treatment (52). The IL-17 stimulation alters the uptake pathway of trypomastigotes promoting phagocytosis over active invasion of macrophages. Phagocytosis of trypomastigotes enhances the number of internalized parasite and prolongs its residency in the endosomal/lisosomal compartment. A recent report demonstrates that IL-17-mediated direct protection of infected macrophages involves the NADPH oxidase activation and ROS production (53). In concordance, mice deficient in IL-23 (IL23p19KO), a potent inducer of IL-17A production, show enhanced parasitemia and a greater susceptibility to acute *T. cruzi* infection compared with infected WT mice. Moreover, IL-17AKO mice seemed to be even more susceptible to *T. cruzi* infection than IL23p19KO mice. The fact that the treatment of macrophages with IL-17A plus IFN-γ is more efficient to eliminate the parasite compared with single-cytokine stimulation, might explain the higher mortality ratio in IL23p19KO and IL-17AKO mice even though the Th1 immune response, measured by IFN-γ production and the iNOS expression, is not impaired during the infection in both KO mice (52). Finally, correlation analysis in Chagas disease patients demonstrates that high IL-17 serum levels are associated with better cardiac function (54–56).

After reaching the cytosol in macrophages, *T. cruzi* is recognized by innate receptor NLRP3, a member of nod-like receptor family (57). Infected mice deficient in IL-1β receptor, ASC, or caspase-1 are defective in NO production and exhibit an enhanced cardiac parasitism and mortality (58). Furthermore, the weak IL-1β production in NLRP3KO macrophages is compensated by an enhanced ROS production that mediates an effective parasite killing, indicating that NLRP3 modulates NADPH oxidase/ROS levels (59). Moreover, these low levels of IL-1β and the partial survival of infected NLRP3KO mice suggest that another Nod-like receptor might be activating inflammasome during infection.

Following cardiac injury, immune cell infiltration and fibroblasts migration trigger tissue remodeling, that is largely dependent on matrix metalloproteinases (MMPs) activity. Immunohistochemistry analysis revealed that vascular wall and infiltrating leukocytes are the cellular source of MMP-2 and MMP-9, which are increased in cardiac tissue during the acute phase of *T. cruzi* infection. As expected, treatment with MMP inhibitors reduces myocarditis in infected mice (60). In Chagas disease patients, T cells, neutrophils and monocytes are source of MMP-2 and MMP-9. *In vitro* stimulation with *T. cruzi*-derived antigens induces higher MMP-2 and MMP-9 expression in monocyte from patients with cardiac clinical forms of Chagas disease compared with those from non-infected patients (61). Recent reports showed a significant positive correlation between the levels of MMP-9, IL-1β and TNF, but a negative correlation between MMP-2 levels and these inflammatory cytokines and a positive correlation with IL-10 expression in serum from chronic Chagas disease patients (61, 62). These results suggest that MMP-9 may be related to inflammation and cardiomyopathy development; meanwhile, MMP-2 would be associated with regulation of chronic inflammatory process. Other study indicated that MMP-2 and MMP-9 levels peaks in plasma from patients clinically asymptomatic with an abnormal ECG (an early indicator of cardiac disease) and progressively augmented in patients with advancing Chagas cardiomyopathy. Although MMPs are strongly associated with several inflammatory diseases, the data suggested that MMP-2 and MMP-9 plasmatic levels could be used as early biomarkers of cardiac disease (63).

It is widely accepted that *T. cruzi* modulate apoptosis of different target cells in order to evade the immune response. In this sense, Freire-de-Lima and coworkers demonstrated that phagocytic removal of apoptotic T lymphocytes by infected-macrophages exacerbate parasite replication by driving its differentiation toward an M2-like phenotype concomitant with TGF-β, IL-10, and PGE2 production (64, 65). TGF-β promotes in macrophages the L-arginine metabolism by arginase to synthesize polyamines which functions as a growth factor for parasites (66). Another work found that blocking apoptosis of CD8^+^ T cells in cocultures, infected macrophages increase NO levels and M1 features leading to restrict intracellular parasite growth (67). In addition, while coculture of apoptotic neutrophils induce an M2-like phenotype in infected macrophages, live neutrophils *via* elastase induce M1 macrophages with NO and TNF production that allow to control *T. cruzi* replication (68). Further studies in human cells are needed to validate these findings.

Similar to others cardiac pathologies, cardiomyocyte apoptosis seems to be determinant of Chagas disease progression. In this sense, it was found that infection induces apoptosis of cardiomyocytes (69, 70). In contrast, several reports demonstrated that *T. cruzi* induces anti-apoptotic effect on cardiac cell (51, 71–75). Indeed, it is plausible to think that cardiac cell exposure to pro-inflammatory milieu may precondition the heart tissue to protect cardiomyocytes from a massive apoptosis (76). This hypothesis is clinically supported by experiments performed by Metzger et al. The authors described that apoptotic cells are increased in the myocardium from Chagas disease patients with severe heart failure. However, the apoptotic cells are not cardiomyocytes, and the majority of TUNEL^+^ cells are also CD68^+^ (human macrophage marker) (77), similar results also being observed by other researchers (78). Related to this, an intense Bcl-2 expression is induced in cardiomyocytes during the acute phase of the experimental infection, likely establishing a higher threshold to apoptosis in this cell type (73).

Although enthusiastic researchers have suggested the therapeutic use of apoptosis inhibitors as an attractive choice for adjuvant therapy during the chronic phase (79), the effects of apoptosis modulation on the outcome of chronic cardiomyopathy requires further studies.

#### Viral Myocarditis

The cardiotropic viruses that cause myocarditis in humans include enteroviruses, adenoviruses, influenza viruses, cytomegaloviruses, parvoviruses, and herpes viruses. Although most of these viruses commonly cause only mild upper respiratory or gastrointestinal complications in most individuals, a few infected people develop cardiac clinical symptoms (80). The enteroviral coxsackievirus B group type 3 (CVB3), adenoviruses parvovirus B19, and human herpes virus 6 are frequently observed in myocarditis biopsies. The most common viral myocarditis is caused by CVB3 infection. It affects about 5–20% of world population, and yet lacks efficient treatments. Although acute viral myocarditis is self-limiting in most individuals, in others develop chronic myocarditis, progressive cardiac fibrosis, dilated cardiomyopathy, heart failure, and even death (81–83). Strong clinical and experimental evidence has demonstrated that two main mechanisms are involved in pathogenesis of CVB3-induced myocarditis, first a direct viral injury to cardiac cells during the acute stage of infection and second, the excessive pro-inflammatory immune response against CVB3 which can subsequently evolve to a chronic autoimmune cardiac pathology in some susceptible patients (80).

Monocytes/macrophages represent one of the major infiltrating inflammatory cells in CVB3-induced myocarditis. It was reported that cardiac CCL2 levels are significantly enhanced at 1 dpi and peak at 4 dpi and hence closely involved in CVB3-induced myocarditis initiation, by attracting monocytes. CCL2 incidence in viral myocarditis was even more evident after blocking CCL2 activity *in vivo*, since those infected mice exhibit reduced myocardial cell infiltration, decreased serum CK-MB levels and increased host survival (82).

Other study showed that *in vivo* CVB3 infection also triggers the production of galectin-3 (a β-galactoside binding lectin) in cardiac macrophages during acute and chronic phases. Abrogation of galectin-3 expression or its pharmacological inhibition does not affect viral titers but reduce acute myocarditis and chronic fibrosis, suggesting a critical role of galectin 3-producing macrophages in the induction of fibrosis subsequent to CVB3 infection.

Studies in experimental murine infection with CVB3 have been widely employed as a model of human enteroviral infection and they have significantly contributed to the understanding of the immunological mechanisms underlying acute and chronic viral inflammatory heart disease. Strikingly, male, but not female, mice infected with CVB3 present a severe myocarditis with a pathological process resembling human disease. Although similar viral infection titers have been detected in patients of both genders, the estimated incidence of myocarditis in men is twofold increase or more than in women (84, 85).

Experimental approaches assessing the differential susceptibility against CVB3 infection between male and female mice have greatly contributed to the knowledge of the etiopathological mechanisms of this viral myocarditis. During the early acute infection, cardiac tissue from male and female mice presents high levels of IFN-γ and IL-4, respectively. Consistently, male cardiac macrophages express M1 markers; meanwhile, female cardiac macrophages polarize toward M2 phenotype (86, 87). Employing adoptive transfer experiments of *in vitro* IFN-γ-induced M1 macrophages, the authors demonstrated this pro-inflammatory subset is responsible for myocardial inflammation and damage after CVB3 infection (86).

It is important to highlight that IL-10 has a protective role during virus-induced cardiac fibrotic process by inhibiting fibroblast collagen synthesis (85, 88). As counterpoint, male infected mice exhibit a reduced frequency of cardiac monocytic-myeloid-derived suppressor cell (MDSC) subset and regulatory T cells, which negatively correlates with the severity of cardiac fibrosis induced by CVB3 infection. Although it was reported that myocardial infiltrating immune cells have a different cell composition in both mouse genders, how this difference affects macrophage functions and the development of CVB3-induced myocarditis needs further studies. In this sense, infiltrating NK cells could have a role in the physiopathology of the disease. Kinetic studies of myocardial infiltrate revealed that NK cells arrive together with macrophages and the synergistic action of sex hormones and CVB3 infection contribute to NK cells differential cytokine production (87).

On the other hand, it was reported that CVB3 can efficiently induce endoplasmic reticulum (ER) stress in infected cardiomyocytes and, in turn, stressed myocardial cells transfer the ER stress to macrophages *via* some unknown soluble molecules and facilitate the pro-inflammatory phenotype. In concordance, an *in vivo* pharmacological treatment with ER stress inhibitor induces a substantial reduction of pro-inflammatory cytokines in macrophages, diminishes CK-MB serum levels, relieves myocardial inflammation and improves cardiac functions. Moreover, adoptive transfer experiments of ER stress-inhibited macrophages into infected mice also alleviate viral-myocarditis together with an increase in mice survival, confirming the pathological role of ER stressed macrophages in CVB3-induced myocarditis (89). Other work showed that CVB3 infection in cardiac cells induces ROS production and alters potassium efflux, triggering NLRP3 inflammasome activation in infected cardiomyocytes (90). Therefore, all these studies indicate that the cardiac environment that shape macrophages polarization has a key role in the outcome of CVB3-induced heart disease.

#### Bacterial Infections

Mononuclear phagocytic cells are essential in cardiac immunity to several myocarditis-related to bacterial infections. Lyme disease is a long-term infection caused by *Borrelia burgdorferi* spirochetes. Its most severe complications are myocarditis and inflammatory arthritis of the lower bearing joints. An important difference between arthritis and myocarditis is the distribution of phagocytes within the lesions; while polymorphonuclear leukocytes are more prevalent in the joint, macrophages are the predominant infiltrating cells in infected hearts (91). The severity of Lyme myocarditis is controlled by CD11c integrin, because CD11cKO mice caused increased cardiac MCP-1 production and consequently, increased macrophage infiltration. The loss of this integrin generates an impaired macrophage immune response leading to an aggravated Lyme disease (92). Similarly, in absence of CCR2, the receptor that induces monocyte recruitment to the site of infection, resistant CCR2KO mice had augmented bacterial heart burden compared with WT counterpart (C57BL/6) suggesting a reduced clearance of the bacteria. In contrast, deficiency of CCR2 in sensitive mice strain CCR2KO (C3H) produced severe inflammation with increased presence of polymorphonuclear cells but decreased *B. burgdorferi* burden in comparison with WT (C3H/HeJ) mice. The less efficient spirochete clearance from hearts of WT C3H mice compared with CCR2KO C3H mice suggests an impaired recruitment or function of macrophages in C3H mice, which may contribute to the susceptibility of this animal strain to *B. burgdorferi* infection (93). Nevertheless, there were no differences in cardiac macrophage polarization of C3H sensitive strain, with similar number of M1 and M2 subtype macrophages at the peak of inflammation (94).

Regarding cytokine production, there are several reports showing that IFN-γ is a key cytokine that modulates the immune response to *B. burgdorferi*, particularly the macrophage activation and functions. Sabino and coworkers proposed that IFN-γ influence the composition of cardiac leukocyte infiltrates. They found that *B. burgdorferi* and IFN-γ synergistically enhance the secretion of mononuclear cell chemoattractants such as CXCL9 and CXCL10, decrease those for neutrophils (CXCL1 and CXCL2), and, in consequence, this modifies the nature of the cellular infiltration (95). Additionally, the production of IFN-γ by iNKT cells enhance the bacterial recognition and activation of macrophages as evidenced by augmented TNF and IL-6 production, leading to an increased phagocytic activity (91). Importantly, IFN-γ is produced in lesions of Lyme patient myocarditis and its levels positively correlate with the severity of the pathology.

Considering innate receptors, it was reported that mice deficient in the intracellular adapter molecule myeloid differentiation antigen 88 (MyD88KO), which is required for several TLR-induced inflammatory responses had 250-fold higher pathogen burden than WT mice. In agreement, *in vitro* experiment confirmed that MyD88KO macrophages phagocytize spirochetes but degrade them more slowly than WT macrophages (96). In accordance, Hawley et al. demonstrated that CR3KO-mediated spirochete internalization requires the participation of CD14 molecule as an accessory receptor and showed that CR3/CD14-mediated phagocytosis generates a pro-inflammatory macrophage phenotype in response to bacterial invasion (97). On the other hand, several reports demonstrate key roles for the intracellular innate receptor Nod2. In *B. burgdorferi* infection context, Nod2-deficient mice resulted in increased myocarditis compared with control mice. Although Nod2 induces inflammatory milieu in *in vitro* models of *B. burgdorferi* infection, BMDM upon a prolonged stimulation of Nod2 no longer respond in the same manner. Indeed, prolonged exposure to the Nod2 ligand results in suppression of pro-inflammatory responses. The authors theorize that the dual role of Nod2 signaling in the inflammatory response may be associated with the chronicity of the infection (98). In the same way, Zlotnikov et al. revealed that a high fat diet-induced obesity (DIO) in a murine model of Lyme disease was associated with systemic suppression of innate immune response. The hearts of DIO-infected mice presented significantly elevated inflammation and increased bacterial burden due to an impaired bacterial uptake and pro-inflammatory cytokine production by macrophages and this effect became more pronounced as infection progressed (99). All these data together substantiate the importance of macrophages in the immune response against this pathogen, but also the dual role that they can play in order to control the infection or perpetuate the inflammation state.

Other bacterial infection in which macrophages play a key role on disease outcome is poststreptococcal myocarditis. *Streptococcus pneumoniae* is capable of invading the heart soon after the development of bacteremia. The pneumococcus is the responsible of the community-acquired pneumonia and sometimes it may worsen by producing the invasive pneumococcal disease that injures the heart. Macrophages have been postulated as inducers of heart-reactive T cells due to their capacity to transfer the inflammatory lesions into normal recipient mice when they are pulsed with group A streptococcus extract (SAE) resulting in an increased serum CK levels, an enzyme that is released in the bloodstream after inflammatory cardiac muscle injury. SAE pulsed macrophages may present to T cells an antigenic determinant characteristic to rheumatogenic streptococci that cross-reacts with a normal component of the heart tissue (100). In accordance, group A streptococcal M proteins had the ability to stimulate human PBMC to proliferate and induce cytotoxic T cells against cultured human heart cells (101). The results suggest a critical role for monocytes/macrophages as antigen-presenting cells in the outcome of heart damage after infection.

More recent studies have proposed that *S. pneumoniae* forms discrete pneumococcus-filled micro lesions in the myocardium during invasive pneumococcal disease (102) generating direct tissue damage. The pneumococci within cardiac micro lesions produce pneumolysin, a cholesterol-dependent pore-forming toxin, which kills cardiomyocytes and macrophages due to pneumolysin-induced necroptosis. This precludes the generation of an effective immune response and perpetuates cardiac damage (103). These reports bring to light that macrophages are crucial for innate immune response to bacterial infections and that the alteration of their functions can alter the outcome of the disease.

### Sterile Inflammation

Although inflammation is the process in which components of the innate immune system respond to an injury or infection, inflammatory response can also be triggered in absence of infection by a process known as *sterile inflammation*. This type of inflammation is implicated in the development of a variety of clinical conditions such as atherosclerosis, cardiac injury, neurodegenerative and metabolic disorders. Unlike microbial-induced inflammation, sterile inflammation is activated by endogenous danger-associated molecular patterns (DAMPs) released as a consequence of cellular injury and/or inflammation, such as oxidized low-density lipoprotein (oxLDL), oxidized phospholipids (oxPLs), amyloid β or uric-acid crystals (104, 105). This sterile inflammatory response induces recruitment of neutrophils and monocytes leading to production of pro-inflammatory cytokines and chemokines, mainly IL-18 and IL-1β in a caspase-1-dependent manner. As mentioned above, monocytes subsets (classical, intermediate, and non-classical) extensively participate in the response to infectious disease; however, their role in sterile inflammation in cardiac ischemia-reperfusion injury and atherosclerosis is still not well understood. Myocardial ischemia-reperfusion injury is a condition that involved cardiomyocytes death affecting contractibility and loss of heart function as a result of a vascular occlusion and loss of tissue irrigation. While different pathways contributed to limit the damage, the inflammatory response in the ischemic heart can be detrimental (106).

The knowledge gained on the origin of resident cardiac macrophages has greatly improved the therapeutic potential of macrophage manipulation in the context of sterile inflammation. Considering that adult mammalian heart contains two macrophage pools which include: Ly6C^low^ MHC-II^low^ and MHC-II^high^ (CCR2^−^) representing embryonically established macrophages and the second pool and much less abundant derived from blood Ly6C^high^ CCR2^+^ monocytes. Altered homeostasis induced by transient depletion (chemically) of monocytes and macrophages population in heart shown proliferation of CCR2^−^ resident macrophages and recruitment of Ly6C^high^ CCR2^+^ monocytes from blood to contribute to tissue macrophages repopulation (107). Thus, after myocardial injury and knowing that NLRP3 inflammasome activation impair tissue regeneration, blockage of CCR2 abolishes ischemic damage. This concept suggests that blockage of CCR2^+^ monocytes influx or recruitment during cardiac ischemia-reperfusion will be protective, while a strategy of depletion of resident macrophages (CCR2^−^) abolished that protection. In the same way, Marchetti et al. applied a pharmacological inhibitory strategy for NLRP3 inflammasome in a mouse model of myocardial injury. They found that in acute myocardial infarction (MI) with reperfusion and in a model of non-ischemic injury induced with doxorubicin, the inhibition of NLRP3 inflammasome cause a significantly reduction of infarct size and preserved systolic function (108).

The understanding of circulating monocytes and cardiac macrophage biology under physiology and disease condition may help discover new mechanisms to target specific subset population of macrophage that impact in cardiac cytoprotective pathways.

### Atherosclerosis

**Figure 4:**
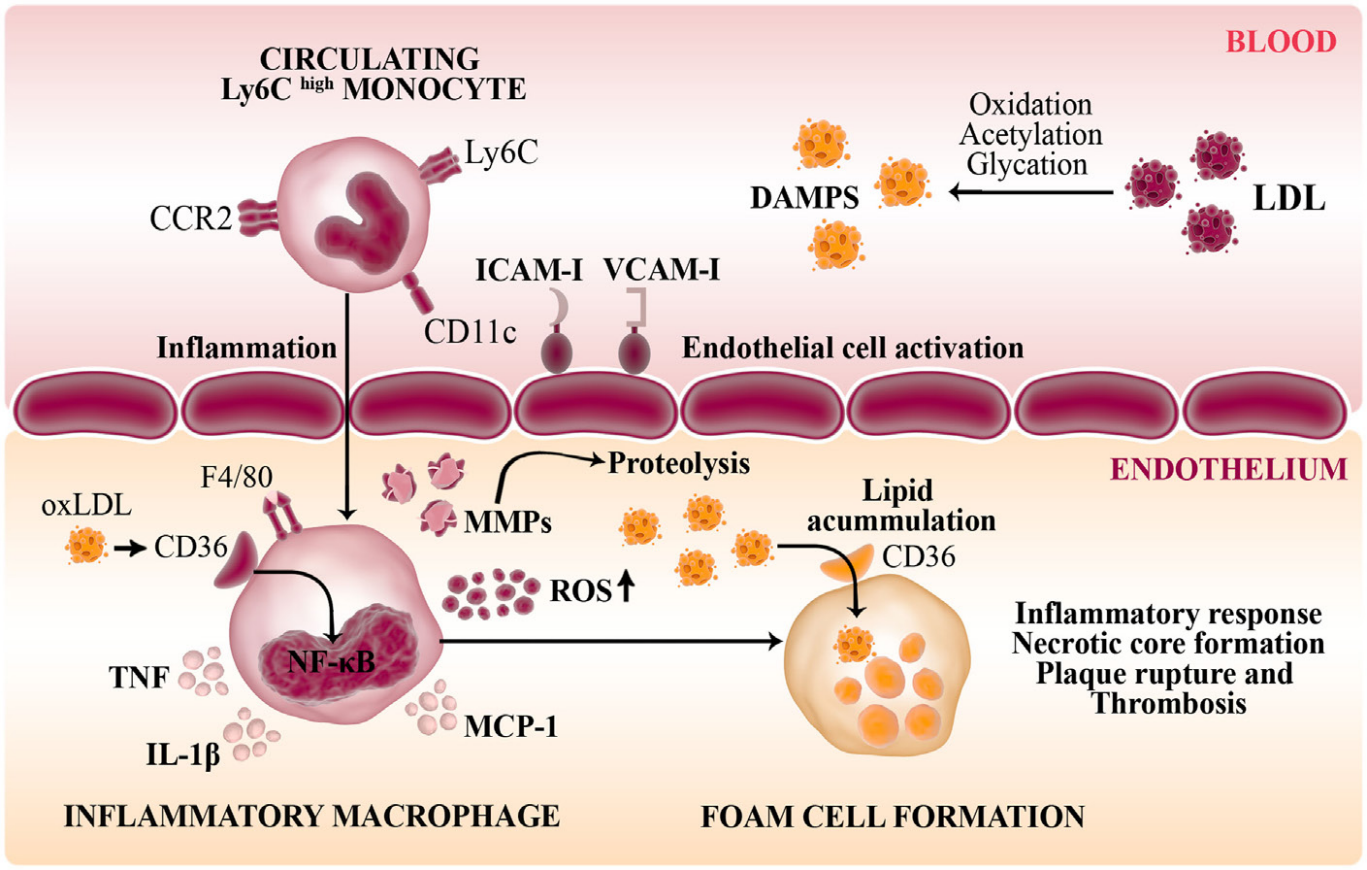
Role of macrophages in the pathogenesis of atherosclerosis. Disturbance of normal vascular physiology is characterized by endothelial activation which stimulates the expression of endothelial adhesion molecules (VCAM-1, ICAM-1) to favor the passage of inflammatory monocytes to the subendothelium. Modified LDL and other DAMPS are recognized by pattern-recognition receptors such as CD36 (scavenger receptor-B) in infiltrating macrophages. Thus, these innate cells are transformed into lipid-laden foam cells in the vasculature intima. The activation of NF-κB in response to ox-LDL induce the secretion of inflammatory cytokines (IL-1β, TNF), NO, MCP-1, and macrophage-derived matrix metalloproteinases (MMPs) that degrade the extracellular matrix generating a maladaptative inflammatory response and contribute to plaque formation.

Atherosclerosis is the result of lipid accumulation and chronic inflammation in the arterial wall where macrophage plays a central role in the development and progression of plaque formation (Figure 4). Thus, transmigration of monocytes into the subendothelial space, lipid uptake (oxLDL) and foam cell differentiation are key step in atherogenesis. The maintenance of a healthy endothelium regulates the precise balance in vascular homeostasis between vasoconstriction and vasodilation, trombogenesis and fibrinolysis, cellular migration and proliferation (109). Disturbance of normal vascular physiology is characterized by endothelial activation, which may be caused by infection or by cardiovascular risk factor such as hyperlipidemia (pathophysiologic). Thus, pro-inflammatory mediators (IL-1β, TNF, and MCP-1) induce a deleterious effect on NO bioavailability increasing ROS which stimulate the expression of endothelial adhesion molecules (VCAM-1, ICAM-1) to favor the passage of monocytes to the subendothelium and promoting their differentiation into macrophages (110).

In atherosclerosis, circulating low-density lipoprotein (LDL) molecules undergoes modifications mainly oxidation, acetylation and glycation that alter the normal metabolism of lipoprotein to promote cytotoxic damage on endothelial cells and macrophages. There is a broad spectrum of mediators that induce LDL oxidation from enzymatic (lipoxygenase and cyclooxygenase) to non-enzymatic reactions (free radicals and oxidants). Some of these reactions have differential selectivity for LDL components as occur with ROS that oxidize predominantly the lipid portion of LDL or the neutrophil myeloperoxidase that oxidize the protein portion (Apo B) of LDL by action of hypochlorous acid byproducts (111, 112).

In this inflammatory milieu, other biomolecules, in addition to LDL, such as lipids and proteins are target of oxidation and nitration reactions generating new species that may affect the fate of the plaque development and limit or exacerbate the inflammatory state. Thus, nitro-fatty acids (NO_2_-FAs) are the product of the reaction of unsaturated fatty acids and nitrogen-derived species (NO and nitrites) (113). Administration of NO_2_-FA in a mice model of atherosclerosis (ApoEKO) exhibited anti-inflammatory and protective actions, decelerating the development of the atheroma plaque, affecting oxLDL uptake and foam cell formation in macrophages (114).

Modified LDLs are recognized and internalized in macrophages by pattern-recognition receptor (PRR) which includes scavenger receptor (SR) and toll-like receptor (TLR). SRs bind diverse ligands and contribute to the clearance of foreign or modified particles. Regarding to macrophage cholesterol metabolism, CD36 (SR-B) and SR-A1 mediate internalization of oxLDL and transform these cells into cholesterol-laden foam cells in the vasculature intima. Exploration in experimental models evidenced that CD36-deficient macrophages exhibited a reduced uptake of oxLDL; further study in ApoE/CD36 double mutant mice described the reduction of the size of atherosclerotic plaque (115, 116). These results emphasized the role of CD36 as a pro-atherogenic receptor in the vasculature and cardiovascular disease. However, discrepancy on CD36 implication as pro- or anti-atherogenic has been raised by other studies in which described advanced atherogenesis in LDLR/CD36 double mutant mice or less macrophage accumulation but increased aortic lesions in ApoE/CD36 double KO (117). The inconsistency of this result may be in part explained by the different experimental model and animal background employed. Besides the discrepancy on CD36 function in atheroma plaque formation the binding of oxLDL stimulates pro-inflammatory signaling pathways (NF-κB) in macrophage contributing to maintain the chronic inflammatory state generated during the onset of atherosclerosis disease.

In atherosclerosis, the recognition of the subsets of recruited monocytes that differentiate into macrophages in the atheroma plaque may be relevant to design new strategies to treat pathological inflammatory process. During the development of atheroma plaque, the recruitment of monocytes are drawn from the Ly6C^high^ circulating subset (precursor of M1 macrophages) but after treatment and control of hyperlipidemia a rapid reduction of plaque inflammation and regression was exhibited, characterized by decrease number of macrophages and the relative increase in markers of M2 macrophages state. Rahman et al. described in a model of aortic arch transplantation that plaque regression is characterized by recruitment of Ly6C^high^ circulating monocytes and the posterior polarization to M2 phenotype (118). This result is contrary to the prevailing paradigm about M2 macrophages recruitment in plaque regression and the authors promote the strategy of M2 macrophage accumulation in atherosclerotic lesions to stimulate plaque regression. Consistent results were obtained in mice treated with IL-13 and IL-4 (promote M2 polarization) which describe protective effects in plaque progression in mice (119).

Summing up, the data obtained clearly illustrate the clue role that mononuclear phagocytic cells play in the development of the atherosclerotic pathology, further molecular mechanisms involved in plaque formation are described in the next chapter.

## Signaling Pathways and Molecules Modulating Cardiovascular Immune Response

### Cytokines and Chemokines

Macrophages are major contributors to the inflammatory response in the setting of cardiovascular diseases. They secrete diverse pro-inflammatory mediators (including cytokines, chemokines, ROS and nitrogen species, among others), and MMPs and they eventually die by necrosis or apoptosis. Dying macrophages release their lipid contents, which lead to the development of a pro-thrombotic necrotic core. The necrotic core is, in turn, a clue component of unstable plaques and contributes to their rupture and the consequent intravascular blood clot that causes MI and stroke. The main effect of cytokines/chemokines released by macrophages is the recruitment of different innate and adaptive immune cell populations into the atherosclerotic lesions (120, 121), thereby amplifying the inflammatory environment. For example, different inflammatory mediators such as IL-1, TNF and IFN-γ increase the expression of MCP-1 in macrophages (120, 122). Also, under inflammatory conditions macrophages produce different enzymes such as MMPs, cathepsins and serine proteases that degrade the extracellular matrix of the atherosclerotic plaque contributing to the pathogenesis of atherosclerosis (123). In this sense, IL-1 and TNF increase the expression of numerous MMPs, such as MMP-9, and their activity contribute to unstable plaque morphology (123).

While initial reports indicated that IFN-γ prevented macrophage foam cell formation (124), subsequent studies demonstrate that this cytokine promotes foam cell formation through diverse mechanisms: enhancing the uptake of oxLDL/AcLDL through SRs (122, 125), diminishing efflux of cholesterol *via* inhibition of ABCA-1 transporter and ApoE expression (126, 127) and increasing ACAT-1 levels (126) in macrophage-derived foam cells. For example, it was recently reported that the axis IFN-γ/STAT1 mediates the uptake of acLDL/oxLDL by human macrophages in an extracellular signal-regulated kinase (ERK)-dependent signaling (125). The activity of IFN-γ as a pro-foam cell mediator has been supported by several *in vivo* studies (128).

Since IFN-γ was identified as a pro-foam cell cytokine, different authors evaluated the effect of other pro-inflammatory cytokines. Studies using a murine macrophage cell line have shown that TNF inhibits the expression of SRs and the uptake of oxLDL (129). Regarding additional mechanisms, more recent evidence has suggested that TNF promotes foam cell formation *in vitro* by reducing the mRNA expression of ABCA-1/ABCG-1 (130), and intracellular lipid catabolism (131), and by enhancing the expression of ACAT-1 and the accumulation of cholesteryl ester (132). However, the role for TNF remains controversial since other studies have shown that TNF enhances ABCA-1 expression and the efflux of cholesterol in peritoneal macrophages stimulated with ApoA–I (133, 134) and since evidences from studies performed in murine models are also contentious (128).

Regarding anti-inflammatory cytokines, it has been demonstrated that TGF-β decreases CD36/SR-A expression and the uptake of oxLDL in human macrophages (135, 136). Additional data emphasized this anti-foam cell effect by showing that TGF-β downregulates CD36/SR-BI mRNA levels in peritoneal macrophages (137) and increases ApoA–I and HDL-stimulated cholesterol efflux and ABCA-1 gene transcription in foam cells (138). Argmann et al. (139) demonstrated that TGF-β1 decreases LPL expression/activity and cholesteryl ester accumulation with a coincident increase in the expression of ABCG-1 in murine macrophages. Recently, it was reported that TGF-β diminishes LPL gene transcription (140) and increases the expression of ApoE (141). Moreover, other group has shown that CD4^+^ CD25^+^ regulatory T cells use TGF-β1 to inhibit the uptake of oxLDL and the expression of CD36/SR-A mRNA (142). These mechanisms evidence an anti-foam cell role for TGF-β1, although it is important to stress that TGF-β may promote development either of anti-atherosclerotic regulatory T cells or of T-helper 17 (Th17) cells, depending on factors in the local milieu. Therefore, TGF-β signaling in T cells could promote stabilization of atherosclerotic plaques through an IL-17-dependent pathway.

The effect of IL-10 on plaque formation has also been reported. This anti-inflammatory mediator downregulates the CD36 SR expression (143) and, in consequence, decreases the accumulation of cholesterol in human macrophages (144). In addition, two independent groups have reported anti-foam cell activity of IL-10. Indeed, they found that IL-10 enhances the uptake and efflux of cholesterol by human macrophages *in vitro* and reduces foam cell formation and atherosclerotic plaque progression *in vivo* (145, 146). Altogether, the results suggest that IL-10 counteract atherogenesis.

Besides M1 and M2 macrophage profiles, additional phenotypes have been described within atherosclerotic lesion and they have become a topic of increasing relevance. In areas of hemorrhage, macrophages respond adaptively to heme acquiring an activation status that was designated hemorrhage-associated macrophages (Mhem). *In vitro* human blood-derived monocytes are induced to Mhem by stimulation with hemoglobin–haptoglobin complexes and it is dependent on an autocrine action of secreted IL-10 (147, 148). This macrophage profile prevents foam cell formation and has antioxidant functions. Other study showed that oxPL products that accumulate in atherosclerotic lesions induce another phenotype designated as Mox. Data indicate that Mox macrophages are characterized by high expression of heme oxygenase-1 and it comprises approximately 30% of all macrophages in established atherosclerotic lesions in experimental models (149). In addition, CXCL4, a chemokine released by activated platelet, was demonstrated to prevent monocytes/macrophages apoptosis and promote its differentiation into a phenotype called “M4” (MMP7^+^ S100A8^+^ CD68^+^). M4 macrophage was described in human coronary atherosclerotic plaques *ex vivo* and postmortem (150, 151). The prevalence of this macrophage subset is higher in patients with severe coronary artery disease and there is a significant correlation between the accumulation of M4 macrophages within the intima and plaque destabilization, which is independent of the overall number of macrophages in the vascular wall (150).

Recently, several groups have published that IL-17A-stimulated macrophages are suggested to constitute a new macrophage population. Barin et al. (152) showed that IL-17A induces activation of primary macrophages in a unique profile of cytokines and chemokines, including IL-12p70, GM-CSF, IL-3, IL-9, CCL4 and CCL5, that can be distinguished from previously characterized macrophage activation states. Moreover, another work showed that IL-17A induces a pro-inflammatory transcriptome in human monocytes/macrophages, including cytokines such as IL-1α and IL-6, chemokines like CCL2, CCL8, CCL20, CXCL1, CXCL2 and CXCL6, genes involved in oxidative stress, upregulation of CD14, CD163 levels and downregulation of genes associated with T cell costimulation. By comparing the entire transcriptomes of M1, M2 and M4 macrophages, authors confirm that IL-17A-treated macrophages are a unique polarization subset (153). On the other hand, it was reported that IL-17 also can strongly amplifies human monocytes/macrophages differentiation toward M2c profile, making it resistant to apoptosis and promoting its MerTK-dependent clearance of apoptotic bodies (154). M2c cells (CD14^bright^ CD16^+^ CD163^+^ MerTK^+^) are a subset of alternatively activated macrophages induced by M-CSF plus IL-10 or glucocorticoids and they are involved in inflammation resolution through phagocytosis of early apoptotic neutrophils and anti-inflammatory cytokines production (155). In contrast with the mentioned effects of IL-17, authors demonstrated that both IFN-γ/Th1 environment and IL-4-chronic exposure impair glucocorticoid and IL-10-induced M2c macrophage differentiation and also alters phagocytosis function of stablished M2c macrophages by reducing MerTK levels and promoting its apoptosis. Thus, this work indicates that Th17 environment orchestrates the resolution of innate inflammation through expansion of M2c regulatory macrophages.

An important point to note is that in a predominantly Th17 environment, monocytes/macrophages have a key role in the IL-17-induced inflammatory responses. In an experimental murine autoimmune myocarditis (EAM) model, the genetic ablation of IL-17RA alters the recruitment of monocytes/macrophages, which is the most numerous component of the inflammatory infiltrate and it is implicated in the cardiac damage (152). In concordance, other work shows that IL-17A promotes cardiac infiltration of Ly6C^high^ monocytes by inducing fibroblasts to produce high levels of cytokines and chemokines. Particularly, GM-CSF production by IL-17-stimulated cardiac fibroblasts drives differentiation of infiltrating monocytes into an even more inflammatory phenotype which further intensifies the myocarditis. Experiments performed in IL17RAKO mice or depletion of Ly6C^high^ monocytes/macrophages demonstrates the involvement of these innate cells in the inflammatory dilated cardiomyopathy (156). On the other hand, in an advanced atherosclerotic model in ApoEKO mice, *in vivo* IL-17A blocking markedly prevents atherosclerotic lesion progression and improves its stability by reducing inflammatory burden and monocyte infiltration (153). Moreover, the CCL2 and CCL5 production were significantly reduced in those IL-17A mAb-treated mice. *In vitro* experiments demonstrated that IL-17A induces adhesion of human monocytes, adhesion and rolling of platelets on endothelial cells. Other studies performed in early stages of atherosclerosis demonstrate that IL-17/IL-17RA axis has a pro-atherogenic role by promoting early monocyte recruitment into plaque through CCL5 production, endothelial VCAM-1 expression and increasing CXCL1 expression on the vessel lumen (157–159).

### Purinergic Signaling

Purinergic system has been recognized as a main pathway to regulate immune response and it is critical to prevent the collateral damage produced by the exacerbation of the immune response in several diseases including cardiac pathologies. In normal conditions, ATP is localized intracellularly where it is the main source of energy for cell functions and it accomplishes indispensable functions in cellular metabolism such as proliferation, migration, motility, biosynthesis and contraction functions within the cardiomyocytes. In addition to its metabolic function, ATP also acts as an important signaling molecule when it is released to the extracellular compartment. After it is released, the extracellular ATP hydrolysis is governed by the activity of two membrane ectoenzymes, the CD39 that catalyzes the phosphohydrolysis of ATP to ADO monophosphate (AMP) and CD73 that hydrolyzes AMP into ADO and inorganic phosphate (45). CD39 and CD73 expression on immune and vascular cells and their enzymatic activities play decisive role in the regulation of the magnitude and duration of purinergic signals that modulate cellular function in an autocrine or paracrine manner (160). These effects are mediated by two types of nucleotide/nucleoside receptors: P1 receptors activated by extracellular ADO and P2 receptors activated by ATP and other nucleotides such as ADP and AMP. The first group includes 4 ADO receptors (ADORA 1, ADORA 2A, ADORA 2B, and ADORA 3), and the second group includes several P2X and P2Y subtypes of receptors (161).

Purinergic signaling is highly described in tumoral context (162) and in autoimmune diseases (163) but poorly studied in the context of cardiac pathologies. Given the high production rate of ATP and the turnover required to maintain its continuous mechanical work, disruption of heart tissue generates the release of high amounts of ATP to the extracellular space. Bonner et al. have reported that cardiomyocytes and erythrocytes do not detectably express the ATP catabolic machinery, but coronary endothelial cells were highly positive for CD39 under basal conditions and a low proportion of these cells express CD73. Furthermore, they stated that resident cardiac APCs and monocytes were the responsible of the first step of ATP degradation in the heart after ischemic injury because they were highly positive for the CD39 while CD73 was absent. Likewise, the dephosphorylating step of AMP to immunosuppressive ADO seems to take place on T lymphocytes and granulocytes because CD73 was mainly expressed in those populations (164).

In contrast to the pro-inflammatory action of ATP, ADO exhibits diverse immunoregulatory roles. Bonner and coworkers found that macrophages stimulated through ADORA 2A and ADORA 2B receptors with ADO undergo M2 phenotype in infarcted mice. In addition, in CD73KO mice there was augmented expression of microbicidal M1 genes, while the expression of M2 genes like arginase-1, IL-10, and TGF-β decreased. This monocyte imbalance toward inflammatory Ly6C^high^-expressing monocytes in mice lacking CD73 had been related to adverse myocardial healing and ventricular dilatation after MI (165). Furthermore, Haskó et al. reported that ADO diminish the oxidative burst by downregulating NO production and inhibit TNF release from monocytes and macrophages (166). A parallel mechanism has also been reported by Cain group in murine isquemia/reperfusion model where ADO pretreatment decreased myocardial TNF production (167). There is strong evidence that ADO can modulate leukocyte adhesion to vascular endothelium *in vivo*. In this sense, Koszalka et al. demonstrated that ADO is an important endogenous pathway to modulate the inflammatory vascular response. After a period of ischemia-reperfusion, they observed significant increase of leukocyte adherence to the vascular endothelium only in the CD73 mutant (168). It has also been suggested that purinergic signals play a role in cellular migration. Analysis of atherosclerotic lesions of P2Y6 KO mice revealed fewer macrophages, diminished RNA expression of IL-6 and VCAM-1, suggesting that deficiency in this ATP receptor limits atherosclerosis and plaque inflammation (169). Recently, it was reported that lack of P2X7 receptor resolute plaque destabilization by inhibiting inflammasome activation and consequently ameliorates experimental atherosclerosis. ATP binding to P2 receptors is crucial to NLRP3 assembly and the subsequent IL-1β release. P2X7 null mice had decreased amount of macrophages in the wound and, in consequence, smaller and less inflamed atherosclerotic lesions than respective control animals (170). Altogether, the data indicate that purinergic signaling can modulate macrophage polarization and infiltration; and that ADO is a key metabolite in suppressing inflammation following injury or infection.

In addition, ADO induces the production of vascular endothelial growth factor (VEGF) in human macrophages, associated with increased hypoxia inducible factor-1α (HIF-1α) expression, the main transcriptional inducer of VEGF in hypoxic milieu. VEGF promotes blood vessel formation, induces cell proliferation and initiates immune cell migration to stimulate vasculogenesis and angiogenesis (171). On the other hand, extracellular ATP contributes to tissue repair by the stimulation of P2X7 receptor and release of the pro-angiogenic factor VEGF (172). Summing up, activation of purinergic receptors in macrophages can promote wound healing and may be targeted to improve cardiac repair mechanisms.

### Metabolism

The heart changes its substrate preferences throughout the life, under physiological or pathological conditions. This metabolic flexibility allows it to adapt to environmental changes. However, shifts in fuel selectivity can become detrimental in the pathological heart (173).

Pathological cardiac hypertrophy is a common consequence of several cardiovascular diseases and is a maladaptive response to chronic stress, sometimes resulting in cardiac failure. Pathological hypertrophy involves a shift in fuel metabolism from fatty-acid oxidation to enhanced reliance on glucose. Increased glucose utilization is characterized by upregulation of glucose uptake and glycolysis concomitant with no change or a decrease in glucose oxidation, resulting in uncoupling of glucose uptake/oxidation metabolism. Nowadays, it is accepted that changes in mitochondrial function and cardiac metabolism precede heart dysfunction, indicating that metabolic remodeling is an early event in the development of cardiac diseases.

Recent interest in understanding the impact of these fundamental metabolic changes on immune cell differentiation and function has yielded numerous studies examining the pathways and metabolites involved in driving protective immune responses. A key question that has emerged is whether differential metabolic activity (i.e., use of glycolysis versus mitochondrial respiration) is coincident with differentiation or if it is a direct driver of alterations in immune cell differentiation and function. Classically activated macrophages that promote inflammation utilize glycolysis and glutamine metabolism to generate large quantities of succinate which enhances inflammation and IL-1β production (174). In contrast, macrophages that play a role in tissue healing and inflammatory resolution increase mitochondrial lipid oxidation (175). A key differentiator between these metabolic phenotypes may arise in the mitochondria, where rather than engaging in conventional electron transport, altered electron flow through the succinate dehydrogenase complex promotes ROS generation and inflammation (176). Enhanced glycolysis in coronary artery disease patients drives mitochondrial ROS production, resulting in pyruvate kinase M2 (PKM2) assembly and translocation to the nucleus. It then phosphorylates and activates STAT3 to promote the production of the cytokines IL-1β and IL-6 (177). Indeed, the increased activation of glycolytic pathway was associated with epigenetic changes in monocyte-derived macrophages describing a new mechanism to explain trained immunity in human innate immune cells (178).

Diabetes is a major risk factor for cardiovascular diseases (179). As such it may lead to heart failure indirectly, by promoting the development of coronary artery disease. However, it is now known that diabetes may cause heart failure also by eliciting a direct detrimental impact on the myocardium leading to the development of cardiac hypertrophy, diastolic and systolic dysfunction (180). The clinical condition associated with the spectrum of cardiac abnormalities induced by diabetes is termed diabetic cardiomyopathy. High glucose levels and dyslipidemia directly induce the upregulation and secretion of cytokines, chemokines and adhesion molecules in immune cells by modulating multiple signaling pathways that converge toward NF-κB signaling (181–183). Activation of the renin–angiotensin–aldosterone system and accumulation of advanced glycation end-products (AGE) and DAMPs molecules like extracellular high-mobility group box-1 protein (HMGB1), also represent key mechanisms that mediate inflammatory response in the diabetic heart primarily by acting TLRs (184–186). Following these initial molecular events, leukocytes infiltrate the myocardium and perpetuate the inflammatory process through secretion of cytokines and pro-fibrotic factors and by increasing the production of ROS. Mediators resulting from this inflammatory cascade, in turn, modulate specific intracellular signaling mechanisms in cardiac cells causing hypertrophy, mitochondrial dysfunction, ER stress and cell death fibroblast proliferation and collagen production. In addition, inflammatory factors may affect myocardial metabolic processes and interfere with cardiomyocyte contractile properties. These abnormalities together promote the development of diabetic cardiomyopathy.

Diabetes-associated metabolic derangements can directly induce cytokine expression and release from cardiac cells. It was previously shown that hyperglycemia augmented the expression of HMGB1 in isolated cardiomyocytes, macrophages and cardiac fibroblasts, thereby activating the MAPK and NF-κB pathways and by inducing TNF and IL-6 secretion (187). Inhibition of HMGB1 decreased myocardial inflammation and fibrosis in a murine model of type 1 diabetes and protected diabetic animals in response to postinfarction remodeling (187, 188).

An increase in circulating lipids may also contribute to cardiac inflammation in diabetes. Fatty acids activate TLR4 that strongly promotes inflammation through the NF-κB pathway (189). Mice with a TLR4 gene deletion demonstrate improved cardiac function and reduced cardiac intracellular lipid accumulation in response to streptozotocin-induced diabetes (190). Fatty acids induce the subsequent expression of IL-6, TNF and IL-1, which can be reversed by peroxisome proliferator-activated receptor-γ, β/δ (PPAR-b/d) (191). Previous work showed that hyperglycemia and high circulating levels of lipids promote inflammation through the activation of protein kinase C (PKC), which activates MAPK pathway thereby inactivating IkB (192).

The challenge to improve cardiovascular disorders therapy by modulating metabolic pathways must take into account potential off-target effects on other cells and tissues. In these sense, by exploiting the intrinsic capacity of monocytes and macrophages to take up foreign particles, the use of nanoparticle formulations might be one option to achieve targeting specificity (193).

### Oxidative Stress

Oxygen is the molecular source of the production of several molecules that damage vital tissues. Reactive oxygen and nitrogen species (RONS) are continuously produced as by-products of the reaction leading to energy production through the mitochondrial and microsomal electron-transport chains. In phagocytes, the oxidative bursts and enzyme systems such as xanthine oxidase and cytochrome P-450 oxidase are the endogenous sources of RONS. While physiological levels of RONS are crucial for cell function, excessive RONS levels trigger oxidative stress. Oxidative stress may damage clues molecules (proteins, lipids, DNA) and could, eventually, conducted to cell death. To prevent oxidative stress, antioxidants have evolved to protect biological systems against RONS-induced damage. Oxidative stress resulting from uncontrolled production of RONS that exceeds the antioxidant capacity, exacerbate atherosclerosis; since it induces endothelial dysfunction by impairing the bioactivity of endothelial NO and promotes leukocyte adhesion, enhances inflammation and thrombosis.

Numerous reports demonstrated that increased oxidative stress is associated with disturbances in cardiovascular diseases. In this sense, it was reported an overproduction of superoxide anion by cardiac dysfunctional mitochondria or increased production of RONS from non-mitochondrial sources in experimental models of type 1 diabetes (194, 195). The augmented production of RONS induces maladaptive cardiac response causing cardiac cells death contributing to cardiovascular disease (196). An excessive oxidative stress has been associated with increased cardiac cell apoptosis, as was evidenced by TUNEL^+^ cells and caspase 3 activation in *T. cruzi*-infected cardiac tissue (44). In this sense, high oxidative state has been associated with amplified lipid and DNA damage and the consequent cardiac cell injury and death. Therapeutic treatment that promotes RONS regulation have been shown to be effective in reducing cardiovascular dysfunction.

It is known that increased oxidative stress induces modifications of LDL, generating DAMPs that are detected by TLRs on different immune cell populations, mainly monocytes/macrophages. A variety of mechanisms mediated by enzymatic (such as 12/15-lipoxygenase and myeloperoxidase) or no-enzymatic redox reaction (such as RONS) boost LDL oxidation in the artery wall. The level of LDL oxidation modulates the immune system, when cells were treated with low oxLDL, the expression level of CD86, a marker for M1 macrophages, increases compared with that in cells treated with high oxLDL. In contrast, the expression level of the marker for M2 macrophages, CD206, significantly increases in cells treated with high oxLDL compared with that in cells treated with native LDL or low oxLDL. These results indicate that the degree of LDL oxidation affects the differentiation of monocytes into different subtypes of macrophages (197). Further research is needed to assess the functional consequences of oxidation processes on human macrophage behavior.

## Conclusion and Perspectives

The crucial role of mononuclear phagocyte system in homeostasis and inflammation described herein makes it a potential therapeutic target for a variety of cardiovascular diseases. However, most of our current knowledge on monocyte/macrophage phenotypes and functions is derived from studies performed in murine models and, as such, requires clinical translation and validation in human cells. Understanding macrophage activation in such settings using high-resolution, single-cell and deep phenotyping approaches will provide the basis for therapeutic proposals directed to target specific subsets while sparing others.

## Author Contributions

LS, NE, NP, RC, GB, and MA wrote the revision. NE and MA design the figures. NE helped to perform the figures. LS and MA critically revised the manuscript.

## Conflict of Interest Statement

The authors declare that the research was conducted in the absence of any commercial or financial relationships that could be construed as a potential conflict of interest.

## Abbreviations

AGE, advanced glycation end-products; APCP, adenosine 5′α,β-methylene-diphosphate; CCR2, chemokine (C–C motif) receptor 2; CD39, ectonucleoside triphosphate diphosphohydrolase-1 enzyme; CD73, ecto-5′-nucleotidase enzyme; CVB3, enteroviral coxsackievirus B group type 3; DC, dendritic cells; HMGB1, high-mobility group box-1 protein; IL, interleukin; iNOS, inducible nitric oxide synthase; KO, knock out; LDL, low-density lipoprotein; LDLR, low-density lipoprotein receptor; Ly6C, lymphocyte antigen 6 complex; Mhem, hemorrhage-associated macrophages; NADPH, nicotinamide adenine dinucleotide phosphate; NF-κB, pro-inflammatory signaling pathways; NO, nitric oxide; NO_2_-FAs, nitro-fatty acids; oxLDL, oxidized low-density lipoprotein; oxPL, oxidized phospholipids; PBMC, peripheral blood mononuclear cells; PKM2, pyruvate kinase M2; PRRs, pattern-recognition receptor; RONS, reactive oxygen and nitrogen species; SR, scavenger receptor; TLR, toll-like receptor; WHO, World Health Organization.
